# Extracts of *Morus nigra* L. Leaves Standardized in Chlorogenic Acid, Rutin and Isoquercitrin: Tyrosinase Inhibition and Cytotoxicity

**DOI:** 10.1371/journal.pone.0163130

**Published:** 2016-09-21

**Authors:** Marcela Medeiros de Freitas, Pedro Ribeiro Fontes, Paula Monteiro Souza, Christopher William Fagg, Eliete Neves Silva Guerra, Yanna Karla de Medeiros Nóbrega, Damaris Silveira, Yris Fonseca-Bazzo, Luiz Alberto Simeoni, Maurício Homem-de-Mello, Pérola Oliveira Magalhães

**Affiliations:** 1 Department of Pharmacy, Health Sciences School, University of Brasília, Brasília, Distrito Federal, Brazil; 2 Department of Botany, Institute of Biological Science, School of Pharmacy, Ceilândia Campus, University of Brasília, Brasília, Distrito Federal, Brazil; 3 Department of Odontology, Health Sciences School, University of Brasília, Brasília, Distrito Federal, Brazil; 4 Immunogenetic and Chronic-degenerative Diseases Laboratory, School of Medicine, University of Brasília, Brasília, Distrito Federal, Brazil; Wageningen Universiteit, NETHERLANDS

## Abstract

Melanogenesis is a process responsible for melanin production, which is stored in melanocytes containing tyrosinase. Inhibition of this enzyme is a target in the cosmetics industry, since it controls undesirable skin conditions such as hyperpigmentation due to the overproduction of melanin. Species of the *Morus* genus are known for the beneficial uses offered in different parts of its plants, including tyrosinase inhibition. Thus, this project aimed to study the inhibitory activity of tyrosinase by extracts from *Morus nigra* leaves as well as the characterization of its chromatographic profile and cytotoxicity in order to become a new therapeutic option from a natural source. *M*. *nigra* leaves were collected, pulverized, equally divided into five batches and the standardized extract was obtained by passive maceration. There was no significant difference between batches for total solids content, yield and moisture content, which shows good reproducibility of the extraction process. Tyrosinase enzymatic activity was determined for each batch, providing the percentage of enzyme inhibition and IC_50_ values obtained by constructing dose-response curves and compared to kojic acid, a well-known tyrosinase inhibitor. High inhibition of tyrosinase activity was observed (above 90% at 15.625 μg/mL). The obtained IC_50_ values ranged from 5.00 μg/mL ± 0.23 to 8.49 μg/mL ± 0.59 and were compared to kojic acid (3.37 μg/mL ± 0.65). High Performance Liquid Chromatography analysis revealed the presence of chlorogenic acid, rutin and, its major compound, isoquercitrin. The chromatographic method employed was validated according to ICH guidelines and the extract was standardized using these polyphenols as markers. Cytotoxicity, assessed by MTT assay, was not observed on murine melanomas, human keratinocytes and mouse fibroblasts in tyrosinase IC_50_ values. This study demonstrated the potential of *M*. *nigra* leaf extract as a promising whitening agent of natural source against skin hyperpigmentation.

## Introduction

Melanin is one of the most widely distributed pigments found in bacteria, fungi, plants and animals [1]. Melanogenesis is initiated with tyrosine oxidation catalyzed by tyrosinase to dopaquinone, which is converted to dopa and dopachrome through auto-oxidation. Dopa is also the substrate of tyrosinase and oxidized to dopaquinone again by the enzyme. The reaction products from dopachrome, dihydroxyindole (DHI) and dihydroxyindole-2-carboxylic acid (DHICA), suffer oxidation to form the brown-to-black eumelanin. In the presence of cysteine or glutathione, dopaquinone is converted to cysteinyldopa or glutathionyldopa subsequently forming the yellow-to-reddish-brown pheomelanin [2, 3]. The mature melanosomes located in the dendrites of melanocytes are then phagocytosed by the surrounding keratinocytes, and it is this process which is responsible for the variety of colors in human skin, hair and eyes [4]. There are two groups of pigmentary disorders: the abnormal presence of exogenous or endogenous pigments in the skin and disorders of the quantitative and qualitative distribution of normal pigment, which includes hyperpigmentation and hypopigmentation [5]. Hyperpigmentation can be induced by external factors such as UV exposure, in which melanin formation and the thickening of the epidermis are the most visible reaction to a natural defense mechanism intended to decrease further penetration to the basal layer where proliferation is taking place [6] and drugs such as certain antibiotics, oral contraceptives, certain antiepileptic agents, chloroquine, levodopa, heavy metals and chemotherapy agents. Internal factors include hormones, sometimes seen during pregnancy, and postinflammatory hyperpigmentation of the skin [7].

Changes in skin pigmentation induce significant cosmetic problems with effect on quality of life [8]. Hyperpigmentation of the skin is a common complaint among patients consulting with dermatologists [9]. The accumulation of an abnormal melanin amount in different specific parts of the skin as more pigmented patches (melasma, freckles, ephelide, senile lentigines etc.) might become an esthetic problem [10]. Melasma has a strong impact on the emotional domain of quality of life, resulting especially from feelings about skin appearance, of which the emotional domain was particularly affected in individuals experiencing a longer duration of this disease [11]. It had long been recognized that the rate of melanin production at baseline and following UV exposure in either cultured pigment cells or intact skin depends on tyrosinase activity, rather than simply on total tyrosinase protein [12].

The enzyme tyrosinase (EC 1.14.18.1), a glycoprotein [13], which catalyzes the aerobic oxidation of tyrosine to produce the pigment melanin, is widely distributed in nature [14], found in plants, insects, mammals and marine animals [15]. Also found in bacteria and fungi, polyphenol oxidases (PPO) or tyrosinases are enzymes of dinuclear copper center active site bound by six or seven histidine residues and a single cysteine residue is highly conserved, being the most well-studied multi-copper oxidase [16, 17].

Use of tyrosinase inhibitors is becoming increasingly important in the cosmetic industry due to their skin-whitening effects, but despite the extensive researches on lightening agents and hyperpigmentation, the existing agents have got limitations in term of high toxicity, low stability, poor skin-penetration, and insufficient activity [18]. Although highly effective, long-term exposure to traditional depigmenting agents, such as hydroquinone, corticosteroids, kojic acid and arbutin can raise several safety concerns with local or systemic side effects such as pigmented contact dermatitis, poor enzyme inhibition, skin irritation and exogenous ochronosis in dark-skinned people. Many plant extracts are more potent inhibitors of melanin formation and are not associated with cytotoxicity or mutagenicity of melanocytes [19–23]. Natural tyrosinase inhibitors are generally considered to be free of harmful side effects and can be produced at reasonable low costs, especially when rich sources are identified [24, 25]. The food industry has been searching for effective antibrowning agents to control discoloration. Antibrowning substances such as cysteine, 4-hexylresorcinol, and kojic acid are well known, however, these chemicals have limited commercial use because of safety problems, cost, and government regulation. In addition, the demand for antibrowning agents from natural products is strong [26].

Moraceae is a family of a flowering plant that comprises about 40 genera and 1400 species [27]. Mulberry (*Morus* sp.) has been domesticated over thousands of years and has been adapted to a wide area of tropical, subtropical, and temperate zones of Asia, Europe, North and South America, and Africa [28]. In 2002, the mulberry (leaf and fruit) was considered not only food, but also drugs, by the Chinese Ministry of Health [29].

*Morus nigra* L. (Moraceae), known as ‘‘black mulberry” or “wild mulberry” belongs to the genus *Morus* and is found in Africa, South America and in Asia, possessing a wide range of medicinal uses and can be used either as single or associated drug to treat different ailments [30]. *M*. *nigra* is used for the treatment of diabetes, cholesterol, cardiovascular problems, obesity and gout [31], it has antimicrobial [32], anticancer [33], hepatoprotective [34, 35] and molluscicidal [30] activities, has a protective action against peroxidative damage to biomembranes and biomolecules [36] and the total flavonoids found in black mulberry fruits possess anti-inflamatory and analgesic effects [37]. In comparative studies, black mulberry fruit has proven to have the highest total phenolic and flavonoid contents when compared to white (*Morus alba* L.) and red (*Morus rubra* L.) mulberry fruits [38], a higher bioactive content due to anthocyanin concentration and antioxidant activity than purple (*M*. *rubra* L.) mulberry genotypes [39]. Similarly, it was demonstrated that black mulberry sugar-free extracts have greater total antioxidant activity and phenolic contents than *M*. *alba* extracts, stating that chlorogenic acid and rutin were found to be the dominant phenolic constituents [40]. Between black, white and Russian (*M*. *alba* var. tatarica L.) mulberry fruits, *M*. *nigra* showed the highest contents of reduced ascorbic acid, titratable acidity, iron, total flavonoids and total monomeric anthocyanins [41]. Due to its edible nature, easy accessibility and economical factor, *M*. *nigra* can be a good source of active compounds [34].

*Morus* species are well-known as plants rich in polyphenols and its extracts have been used as a non-toxic natural therapeutic agent, which also have high potential in applications as skin-whitening agents due to many potent tyrosinase inhibitors being isolated from different parts of the plant [42]. Tyrosinase inhibitors from *Morus* include *M*. *alba* roots [43], leaves [44], twigs and root bark [45]; *M*. *australis* roots [46], dried stems [47] and leaves [48]; *M*. *lhou* stem barks [49] and roots [50]; *M*. *multicaulis* Perr. branch bark [29]; *M*. *notabilis* stem [51] and *M*. *yunnanensis* leaves [52]. Tyrosinase inhibitors were also isolated from *M*. *nigra* stems [53] and roots [54] but to date no reports were found in the literature on tyrosinase inhibition by the extract of *M*. *nigra* leaves, which can serve as a suitable whitening agent as other species of the genus *Morus* have proven to inhibit the enzyme of interest, making this work innovative by suggesting a novel natural skin-whitening source.

Plants naturally produce phytochemicals, which are responsible for different color, flavor, smell, and its natural defense mechanism against diseases [55]. Pure phytoconstituents and plant extracts are rich in chemical diversity, an inexhaustible source for new drug development [56]. Natural products obtained from plants are important to the therapy of various disease conditions [57]. Herbal medicines are in great demand for primary healthcare because of their wide biological activities, higher safety margins and lesser costs. The lack of complete standardization for plant formulations is a great challenge. They are prone to contamination, deterioration and variation in composition due to its natural origin. Therefore, quality control of herbal medicines is often complicated [58]. Quality control begins with the plant material, which is the most important variation factor in manufacturing herbal medicinal products. Plants are inevitably inconstant because their composition may be influenced by multiple factors, such as origin, growth, harvesting, drying, and storage conditions [59].

Due to the importance of standardization of plants to ensure quality control and the uses of *Morus* species in traditional medicine and a high source of natural compounds, the aim of this study was to evaluate the leaf extract of *M*. *nigra* for tyrosinase inhibitory activity, as well as the characterization of the chromatographic profile of its standardized extract identifying compounds responsible for enzyme inhibition, development and validation of a High Performance Liquid Chromatography with UV detection (HPLC-DAD) method aiming to standardize it for chlorogenic acid, rutin and isoquercitrin, and asses the viability of extract incorporation in cosmetics of topical use from its cytotoxicity in cell lines that constitute skin, such as fibroblasts and keratinocytes in order to become a new therapeutic option to whitening agents originated from natural source, which can also be used in the food industry for acting as an antibrowning agent for fruits, vegetables and juices. As far as the authors are concerned, this is the first study that evaluates tyrosinase inhibition and quantifies the presence of these three polyphenols in *M*. *nigra* leaf extract.

## Materials and Methods

### Chemicals and Reagents

Tyrosinase from mushroom (lyophilized powder, ≥1000 unit/mg solid), L-tyrosine (≥98%), kojic acid, chlorogenic acid (≥95%), rutin (≥94%) and isoquercitrin (≥90%) were purchased from Sigma-Aldrich (St Louis, MO, USA). Aqueous ethanol (95%) used for extraction was prepared from absolute ethanol Synth (Diadema, SP, Brazil). Methanol used to dilute samples, acetonitrile used as mobile phase for HPLC analysis and phosphoric acid 85% were purchased from Tedia (Fairfield, OH, USA). Water used to prepare the mobile phase was purified in a Milli-Q-plus System (Millipore, Bedforte, MA, USA).

### Plant Materials

*M*. *nigra* L. leaves were cultivated, identified and collected on March 24th 2014 in Brasilia, DF, Brazil by Professor Christopher Fagg (Botany Department, Faculty of Ceilândia, University of Brasília). The voucher specimen was deposited in the Herbarium of the University of Brasília, number Fagg CW 2302.

### Extraction

Leaves were air-dried, then artificially dried at 40°C until humidity reached 9%. The dried material was powdered (Mesh size 230–35), divided into 5 batches of 210 g and extracted with 1050 mL aqueous ethanol 95% at room temperature for 10 days with daily manual agitation. In order to evaluate the reproducibility of the extraction process, the total solids content was measured in an infrared moisture analyzer (Gehaka model IV2000, São Paulo—SP, Brazil) in samples of 2 mL of filtered ethanol extract from each batch, in triplicate. The ethanol extracts were evaporated to dryness under reduced pressure at 40°C followed by lyophilization, thus obtaining the Standardized Extract of *M*. *nigra* Leaves (SEML), which were stored at -20°C. The humidity content was measured in the infrared moisture analyzer previously described with 1 g of the dried extract from each batch, in triplicate.

### Assay of Tyrosinase Activity

Tyrosinase inhibition assay was performed according to Khatib et al. (2005) [60] with modifications [25]. Sodium phosphate buffer (60 μL, 50 mM) at pH 6.5, 30 μL tyrosinase (250 U/mL) and 10 μL of *M*. *nigra* extract (1 mg/mL) were inserted into 96-well plates. Extracts from all five batches were dissolved in methanol and tested at concentrations ranging from 1000 μg/mL to 0.49 μg/mL. After 5 min of incubation at room temperature, 100 μL L-tyrosine (2 mM) were added and incubated for additional 20 min. The optical density (OD) of the samples were measured in a microplate reader (Beckman Coulter, model DTX 800, Lagerhausstrasse—Austria) at 450 nm and compared to control without inhibitor, demonstrating a linear color change with time during the 20 min of the experiment. Control incubations represent 100% enzyme activity and were conducted in a similar way by replacing extracts by buffer. For blank incubation, in order to eliminate the absorbance produced by the extract, the enzyme solution was replaced by buffer. The inhibitory activity was determined by comparing the enzyme activity in the absence and presence of the evaluated inhibitor. Kojic acid was used as positive control. For negative control the solvents used in the extraction process and to dilute the crude extract, ethanol and methanol, respectively, were tested to confirm there is no interference with tyrosinase inhibitory activity.

### Preparation of standard solution

Standard stock solutions (1 mg/mL) of chlorogenic acid, quercetin-3-O-rutinoside (rutin) and quercetin-3-β-D-glucoside (isoquercitrin) were prepared by solubilization of 10 mg of each reference standard with 10 mL methanol in a volumetric flask. A mixed stock solution of 100 μg/mL was prepared from 1 mL of each reference standard (1 mg/mL) with methanol in a 10 mL volumetric flask, followed by different concentrations of mixed standard solutions in 10 mL volumetric flask (0.075–10.0 μg/mL), for establishment of calibration curves and limits of detection and quantitation for all three polyphenols.

### Sample preparation

Standardized extract of *M*. *nigra* leaves was weighed (10 mg, batch 4) and solubilized in methanol (5 mL) to a final concentration of 2 mg/mL. All samples were filtered in a 0.45 μm, 13 mm Millex (Merck Millipore, Carrigtwohill Co. Cork, Ireland) and injection volume was 10 μL.

### Determination of chlorogenic acid, rutin and isoquercitrin by RP-HPLC

A High Performance Liquid Chromatography (HPLC) was used to determine chlorogenic acid, rutin and isoquercitrin levels in *M*. *nigra* ethanol extract. A Dionex UltiMate 3000 liquid chromatography system equipped with a Diode Array Detector DAD-3000 operating in 280, 330 and 354 nm was used. System was configured with a DGP-3600SD solvent pump unit, WPS-3000 SplitLoop injector and column oven TCC-3200 at 25°C, all from Dionex. Separation was performed in a LiChroCART Purospher STAR RP C18 column (150 x 4,6 mm, 5 μm particle size, Merck, Germany), equipped with a pre-column of same characteristics (4 x 4; 5 μm particle size, Merck, Germany). The mobile phase was 1% phosphoric acid and acetonitrile gradient at a flow rate of 0.5 mL/min, starting at 90% (1% phosphoric acid) decreasing to 70% until t = 40 minutes and returning to 90% until t = 45 minutes. All solvents used as mobile phase were filtered in a 0.22 μm, 47 mm polyvinylidene fluoride (PVDF) membrane (Merck Millipore) and degas in an ultrasonic cleaner (Bransonic, CT, USA). Data were collected extracting chromatograms at 354 nm using the Thermo Fisher Scientific software Chromeleon, version 7.1.2.1541.

The analytical methodology was validated following the ICH guidelines [61] and the validation guide of analytical and bioanalytical methods established by the Brazilian National Agency of Sanitary Vigilance [62]. Validation was performed considering specificity, linearity, accuracy, precision, detection limit, quantitation limit and robustness.

The specificity of the method was verified by analysis of sample degradation products by acid/base hydrolysis induction. Therefore, 10 mg of SEML were weighed and solubilized in 1 mL of hydrochloric acid (1 M) for acid hydrolysis and 10 mg were solubilized in 1 mL of sodium hydroxide (1 M) for basic hydrolysis. These solutions were incubated at 60°C for 60 minutes. After cooling to room temperature, the solutions were neutralized with 1 mL of sodium hydroxide (1 M) and hydrochloric acid (1 M), respectively. After freezing, samples were lyophilized and then methanol (5 mL) was added to a final concentration of 2 mg/mL. HPLC analysis was performed in triplicate. Retention time and peak areas of chlorogenic acid, rutin and isoquercitrin on samples prepared without acid/base hydrolysis induction were compared to the results obtained from samples after acid/base hydrolysis induction.

Linearity was assessed by the linear regression method of three authentic calibration curves, through the analysis of eight different concentrations of chlorogenic acid (0.2, 0.3, 0.4, 0.5, 1.0, 2.5, 5.0, and 10.0 μg/mL) and nine different concentrations of rutin and isoquercitrin standard solutions (0.1, 0.2, 0.3, 0.4, 0.5, 1.0, 2.5, 5.0, and 10.0 μg/mL). The correlation coefficient (*r*), y-intercept, slope of the regression line, and residual sum of squares were obtained using the software GraphPadPrism.

In intra-day precision, six determinations at 100% of the test concentration from the solubilized SEML (2 mg/mL) were analyzed by HPLC on the same day. The inter-day precision was determined in triplicate and analyzed on three consecutive days with the same analyst. The precision is expressed as the percent Relative Standard Deviation (RSD).

Accuracy was assessed using three replicates of three concentration levels through the standard addition of chlorogenic acid, rutin and isoquercitrin to the sample. In order to achieve solutions of low, normal and high concentrations (about 80%, 100% and 120%) of the solubilized SEML, mixed standard solutions (100 μg/mL) of chlorogenic acid (10 μL, 20 μL and 30 μL), rutin (30 μL, 50 μL and 70 μL) and isoquercitrin (60 μL, 100 μL and 140 μL) were prepared adjusting the final volume to 2 mL of methanol. 500 μL of each mixed solution was added to 500 μL of the solubilized SEML (2 mg/mL, 0.99 μg/mL chlorogenic acid; 2.53 μg/mL rutin and 4.84 μg/mL isoquercitrin) resulting in the theoretical final concentrations of 0.75, 1.00 and 1.25 μg/mL for chlorogenic acid, 2.02, 2.52 and 3.02 μg/mL for rutin and 3.92, 4.92 and 5.92 μg/mL for isoquercitrin. Finally, the solutions were analyzed by the developed HPLC method. Accuracy was expressed as percent of recovery, which was estimated as the relation between the experimental concentrations and the theoretical concentrations, using the formula: recovery (%) = 100 x (experimental concentration/theoretical concentration).

The Limit of Detection (LOD) and Limit of Quantitation (LOQ) were determined according to the method based on parameters of the calibration curve, where LOD was expressed as (3.3 x ơ)/*S* and LOQ was expressed as (10 x ơ)/*S*, where ơ is the standard deviation of the response and *S* is the slope of calibration curve.

The robustness of the method was determined by chromatographic analysis of samples under different conditions, such as changes in wavelength (280 and 330 nm), flow (0.3 and 0.7 mL/min) and temperature (35 and 45°C). The effects of the parameters retention time and peak area were observed.

Samples (2 mg/mL) of SEML from batches 1, 2, 3, 4 and 5 were evaluated, in triplicate, against the validated method for quantification of chlorogenic acid, rutin and isoquercitrin and analyzed statistically by ANOVA with Tukey multiple comparison method (*p*<0.05).

### Cytotoxicity Assay

The cell lines, one human keratinocyte cell line (HaCat), one fibroblast cell line (L-929) and one melanoma cell line (B16F10), were grown as monolayers in a mixture of Dulbecco’s modified Eagle medium and supplemented with 10% fetal bovine serum and 1% antibiotics (penicillin-streptomycin). Cells were maintained at 37°C and 5% of CO_2_. For all experiments, cells were detached with trypsin (0.25%)/EDTA (1 mM) solution. All cell culture reagents were purchased from Sigma-Aldrich (St Louis, MO, USA). The melanoma cell line was acquired by Rio de Janeiro Cell Bank—Scientific Technical Association Paul Ehrlich (Duque de Caxias—RJ, Brazil). The human keratinocyte and mouse fibroblasts cell lines used are described in the ATCC (American Type Culture Collection).

The cells were seeded at the density of 5x10^3^ cells/well for HaCat and 1x10^4^ for L-929 and B16F10 in a 96-well plate. After 24 hours, the cells were treated with SEML (batch 4) at concentrations ranging from 2000 μg/mL to 0.98 μg/mL. For negative control cells were treated with 5% ethanol and methanol, solvents used in the extraction process and to dissolve the plant extract, respectively. SDS 10% was used as cell death control. Following 24 hours after extracts’ treatment, cell death was assessed by MTT assay using Cell Counting Kit-8 (CCK-8, Sigma) reagent and the absorbance was measured at 450 nm in the same microplate reader mentioned above. CCK-8 uses WST-8, which is bioreduced by cellular dehydrogenases to an orange formazan product that is soluble in tissue culture medium and is directly proportional to the number of living cells. All experiments were performed in triplicates.

## Results and Discussion

### Reproducibility of the extraction process

The total solids contents found for batches 1, 2, 3, 4 and 5 were 1.70% ± 0.26; 1.83% ± 0.12; 1.57% ± 0.06; 1.53% ± 0.06 and 1.50 ± 0.10%, respectively. The yields of the extraction processes of *M*. *nigra* leaves by 95% ethanol for batches 1, 2, 4 and 5 were 7.82%; 7.35%; 8.52%; 7.68%, respectively. A sample from batch 3 was discarded due to contamination during the drying period, therefore the yield could not be calculated for this batch. The humidity contents found for batches 1, 2, 3, 4 and 5 were 0.67% ± 0.15; 0.60% ± 0.10; 0.70% ± 0.10; 0.76% ± 0.19 and 0.67% ± 0.06, respectively. The data suggest that the profile of the moisture content is directly proportional to the efficiency of the extraction process. The higher the yield obtained, the greater the moisture content of the extract, as observed for batch 4 which has the largest percentage yield (8.52%) and moisture content (0.76%). In contrast, the batch with lowest yield (7.35%) had the lowest moisture content (0.60%, batch 2). The results show no significant difference between batches for total solids content and humidity content in analysis by ANOVA (*p*<0.05) with Tukey method of multiple comparison. It was demonstrated similarity among the five batches of standardized extract of *M*. *nigra* leaves for the parameters evaluated, indicating that the process of obtaining the standardized extract was reproducible.

### Evaluation of tyrosinase inhibition

**Fig 1:**
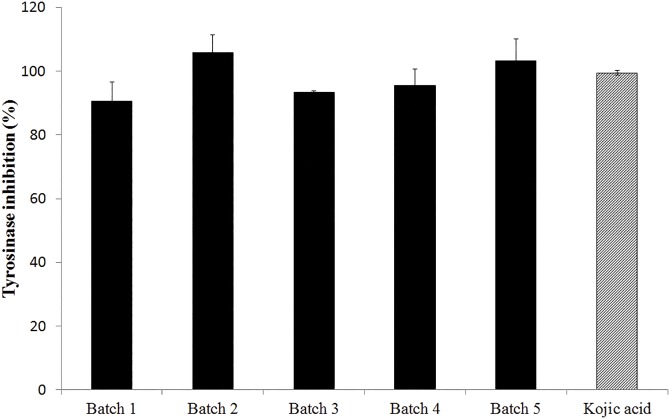
Tyrosinase inhibition activity profile of five standardized extracts of *M*. *nigra* leaves and kojic acid.

To assess the inhibition potential of *M*. *nigra* leaf extract on the enzyme tyrosinase, the five batches were tested and compared against a positive control, kojic acid, a natural agent from the fungus *Aspergillus oryzae*. In order to study the inhibition profile of the extracts on tyrosinase activity the IC_50_ values were determined by the construction of dose-response curves through serial dilution of the extracts and kojic acid at concentrations of 1000 μg/mL to 0.49 μg/mL, using the software GraphPad Prism. The percentages of tyrosinase inhibition were 90.48% ± 6.02; 100.00% ± 5.41; 93.45% ± 0.35; 95.59% ± 5.14 and 100.00 ± 6.70% for batches 1, 2, 3, 4 and 5, respectively. Kojic acid showed tyrosinase inhibitory activity of 99.41% ± 0.78. The data showed that the five batches of *M*. *nigra* were able to inhibit the enzyme tyrosinase, showing an inhibition higher than 90% until concentration of 15.625 μg/mL for all batches, similar to kojic acid (Fig 1).

**Fig 2:**
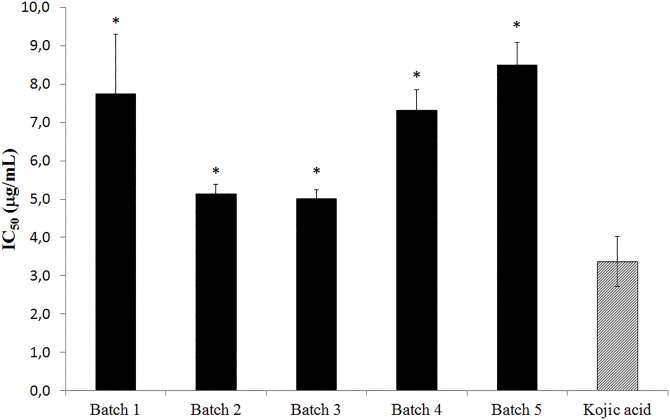
IC_50_ values of five standardized extracts of *M*. *nigra* leaves compared to kojic acid. The significant differences related to the IC_50_ values on tyrosinase inhibition activity (*p<0.05) were calculated by Student's t test.

The calculated IC_50_ values were 7.75 μg/mL ± 1.55; 5.14 μg/mL ± 0.24; 5.00 μg/mL ± 0.23; 7.31 μg/mL ± 0.55 and 8.49 μg/mL ± 0.59 for batches 1, 2, 3, 4 and 5, respectively. Kojic acid obtained IC_50_ value of 3.37 μg/mL ± 0.65. In analysis by Student's t test the five batches of standardized extract of *M*. *nigra* leaves showed significant difference (*p*<0.05) compared to kojic acid (Fig 2).

The solvents used in the extraction process and solubilization of the plant extract to carry out this assay, 95% ethanol and 5% methanol, respectively, showed no tyrosinase inhibitory activity, thus ensuring non-interference with the results in enzyme inhibition. When leaving the microplate to rest for 24 hours a color change was observed from orange to black in wells where there was no enzyme inhibition. The enzyme reaction between tyrosinase and L-tyrosine generates an orange dopachrome, which leads to black melanin (eumelanin) synthesis in melanogenesis pathway [4]. Therefore, it is suggested that the color change is related to eumelanin synthesis. It was observed that kojic acid showed color change in wells of concentrations higher than the IC_50_ values calculated in 30 minutes incubation period (solution in wells turned black in long-term incubation from 0.49 to 62.5 μg/mL), whereas all *M*. *nigra* extracts showed color change in the same wells of concentrations below its IC_50_ values (solution in wells turned black in long-term incubation from 0.49 to 7.81 μg/mL in all batches), indicating that *M*. *nigra* extract is more stable than kojic acid at tyrosinase inhibition, since the positive control was not able to maintain its inhibitory activity over time.

Chang et al. (2011) found that the ethanolic extract of *M*. *alba* L. twigs and ethanolic extract of *M*. *alba* L. root bark exhibited 0–78% and 0–62% inhibitory effects on tyrosinase activity in the range of 0–60 μg/mL [45], while the standardized extract of *M*. *nigra* leaves showed tyrosinase inhibition above 90% at 60 μg/mL concentration. Zheng et al. (2012) found IC_50_ values of 22.53±0.08 μg/mL and 27.88±0.11 μg/mL for *M*. *australis* root and twig, respectively [46], while the standardized extract of *M*. *nigra* leaves showed IC_50_ values from 5.0±0.23 μg/mL to 8.49±0.59 μg/mL.

### HPLC analysis

**Fig 3:**
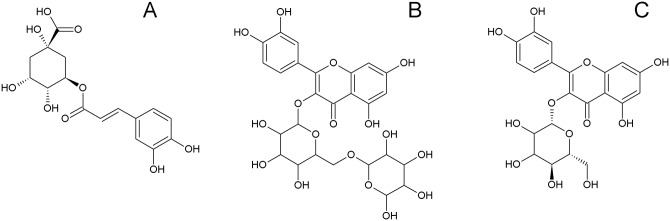
Identified compounds in standardized extract of *M*. *nigra* leaves. (A) Chlorogenic acid. (B) Rutin. (C) Isoquercitrin.

In the present work, HPLC with UV detection and gradient mobile phase mode was proposed as a suitable method for quantitative determination of chlorogenic acid, rutin and isoquercitrin in standardized extract of *M*. *nigra* leaves (Fig 3).

**Fig 4:**
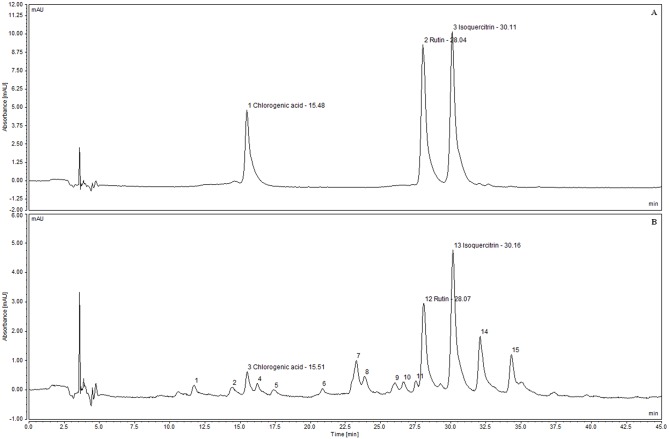
HPLC chromatograms. (A) Reference standards (10 μg/mL). (B) Diluted standardized extract of *M*. *nigra* leaves (2 mg/mL) λ = 354 nm.

Peak areas were used in order to quantify the contents of these polyphenols in SEML. Three linear equations obtained by the linear regression of peak areas *versus* concentrations of the standards were employed, resulting in 0.50, 1.27 and 2.42 mg/g for chlorogenic acid, rutin and isoquercitrin in SEML, respectively. Sanchéz-Salcedo et al. (2016) investigated the fingerprint of polyphenolic compounds in leaves of mulberry clones from *M*. *alba* and *M*. *nigra* which revealed that flavonols were the most relevant and caffeoylquinic acids are the phenolic compounds present in larger quantities in mulberry leaves, being chlorogenic acid (5-caffeoylquinic acid) the predominant one [63]. In a previous study, Sánchez-Salcedo et al. (2015) found that the predominant flavonols in mulberry leaves of *M*. *alba* and *M*. *nigra* species were quercetin-3-O-glucoside (isoquercitrin), followed by quercetin-3-O-(6-malonyl)-β-glucopyranoside = quercetin-rutinoside (rutin) and kaempferol-3-O-(6-malonyl) glucoside. In particular, they ranged from 0.92 to 3.73 mg/g dw for quercetin-3-glucoside, from 0.35 to 1.84 mg/g dw for quercetin-3-O-(6-malonyl)-β-glucopyranoside, between 0.58 to 1.80 mg/g dw for rutin, and from 0.11 to 0.80 mg/g dw for kaempferol-3-O-(6-malonyl)glucoside [64]. Isoquercitrin was found to be the major compound followed by rutin, as shown on Fig 4. In *M*. *alba* 70% ethanol leaf extract, Hunyadi et al. (2012) [65] found 3.58 ± 0.06% of chlorogenic acid, 1.96 ± 0.03% of rutin and 1.20 ± 0.02% of isoquercitrin, chlorogenic acid being the major compound.

**Fig 5:**
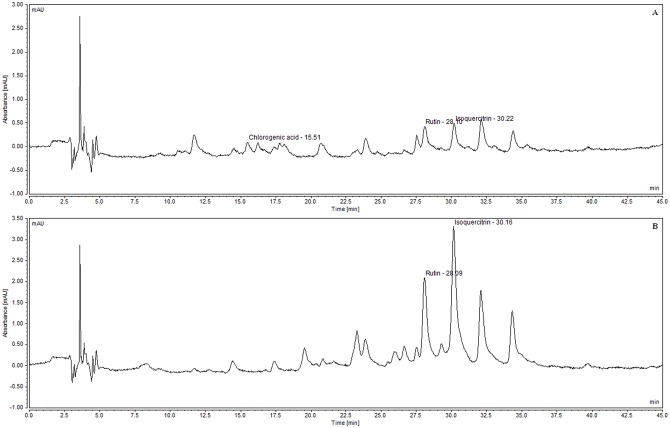
Specificity of the proposed method. (A) Acid hydrolysis and (B) Basic hydrolysis of diluted standardized extract of *M*. *nigra* leaves (2 mg/mL) λ = 354nm.

When undergoing acid hydrolysis (Fig 5A) chlorogenic acid, rutin and isoquercitrin decreased by 80%, 88% and 91% from the average of the measurements taken of their peak areas, respectively, but chlorogenic acid and rutin did not elute in the same retention time of the degradation products generated by acid hydrolysis. Basic hydrolysis (Fig 5B) degraded chlorogenic acid. Flavonoids rutin and isoquercitrin decreased by 54% and 48% from the average of the measurements taken of their peak areas, respectively, but did not elute in the same retention time of the degradation products generated by basic hydrolysis, demonstrating method specificity.

**Fig 6:**
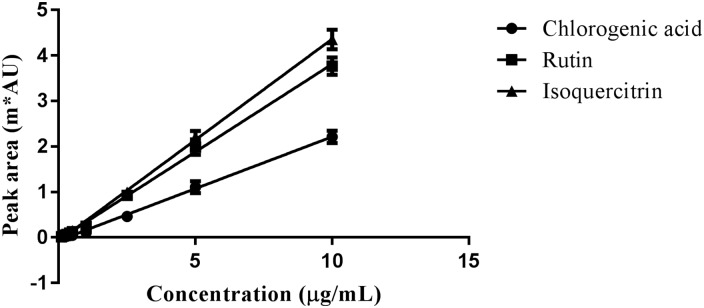
Calibration curves for chlorogenic acid, rutin and isoquercitrin.

The linear equation was calculated from the average absorbance values obtained in the studied concentration range (0.2–10.0 μg/mL for chlorogenic acid and 0.1–10.0 μg/mL for rutin and isoquercitrin). Linear regression was built from three independent calibration curves for chlorogenic acid, rutin and isoquercitrin (Fig 6).

The correlation coefficient (*r*), intercepts, the slope and the residual sum of squares are shown on Table 1. All calibration curves showed satisfying linear regressions within testing ranges, since all correlation coefficients are above 0.99.

**Table 1:** Linearity of chlorogenic acid, rutin and isoquercitrin.

Polyphenol	Concentration (μg/mL)	Correlation coefficient (*r*)	Intercept	Slope	Residual sum of squares
Chlorogenic Acid	0.2–10.0	0.996	-0.0647 ± 0.0176	0.228 ± 0.00431	0.0682
Rutin	0.1–10.0	0.997	-0.0483 ± 0.0215	0.386 ± 0.00560	0.0912
Isoquercitrin		0.998	-0.0746 ± 0.0224	0.444 ± 0.00584	0.0951

In intra-day precision the mean relative standard deviation values found in the analysis of solubilized SEML (2 mg/mL) were of 10.20%, 11.73% and 8.33% for chlorogenic acid, rutin and isoquercitrin, respectively. In the inter-day analysis, variations of 6.99%, 3.59% and 3.99% were found for chlorogenic acid, rutin and isoquercitrin, respectively, as shown on Table 2. The method is precise for all three polyphenols studied once it is in agreement with the minimum requirements stated in the orientation guide for the registration of herbal drugs [66]. According to these rules, values of Relative Standard Deviation (RSD) greater than 15% are not admitted.

**Table 2:** Precision of chlorogenic acid, rutin and isoquercitrin.

Precision	Concentration (μg/mL)	RSD (%)
Chlorogenic acid		
Intra-day^a^	1.15 ± 0.12	10.20
Inter-day^b^	0.99 ± 0.07	6.99
Rutin		
Intra-day^a^	2.89 ± 0.34	11.73
Inter-day^b^	2.85 ± 0.10	3.59
Isoquercitrin		
Intra-day^a^	4.99 ± 0.42	8.33
Inter-day^b^	5.13 ± 0.20	3.99

^a^6 replicates were assayed on the same day;

^b^3 replicates were assayed on different days (RSD = Relative Standard Deviation). The results are mean ± standard deviation.

The mean values (± standard deviation) of the percentage analytical recoveries for the experimental concentration over the theoretical concentration for chlorogenic acid, rutin and isoquercitrin are presented on Table 3. These results indicate that the chromatographic conditions used are reliable to quantify these polyphenols in the range evaluated.

**Table 3:** Accuracy of chlorogenic acid, rutin and isoquercitrin.

Concentration (%)	Theoretical concentration (μg/mL)	Experimental concentration (μg/mL)	Recovery (%)
Chlorogenic Acid			
72^a^	0.75	0.73 ± 0.06	97.49 ± 7.74
95^b^	1.00	0.91 ± 0.06	91.40 ± 5.69
117^c^	1.25	1.08 ± 0.06	86.49 ± 4.50
Rutin			
80^a^	2.02	1.98 ± 0.05	98.44 ± 2.33
99^b^	2.52	2.44 ± 0.15	97.07 ± 6.03
119^c^	3.02	2.86 ± 0.11	95.01 ± 3.81
Isoquercitrin			
81^a^	3.92	4.00 ± 0.07	102.06 ± 1.85
102^b^	4.92	5.06 ± 0.23	102.92 ± 4.64
122^c^	5.92	6.10 ± 0.35	103.09 ± 5.89

^a^Low concentration;

^b^Intermediate concentration;

^c^High concentration. The results are mean ± standard deviation of 3 experiments.

The detection and quantitation limits found for chlorogenic acid, rutin and isoquercitrin were determined based on the parameters of the calibration curve, which contains the compounds of interest at a range of concentrations near the detection limit (0.2–1.0 μg/mL for chlorogenic acid and 0.1–0.5 μg/mL for rutin and isoquercitrin). The estimated concentration values calculated using the standard deviation (ơ) of the intercept for limit of detection were 0.11, 0.12 and 0.10 μg/mL for chlorogenic acid, rutin and isoquercitrin, respectively. The calculated quantitation limits were 0.34, 0.35 and 0.30 μg/mL, respectively. Further evaluation, using the visual method, investigated seven injections of the analyzed polyphenols at lower concentrations of 0.1 and 0.075 μg/mL. It was shown that although chlorogenic acid may not be quantified in these concentrations at a wavelength of 354 nm, the polyphenol can be quantified with precision at 330 nm, wavelength in which it has higher absorption, in the lowest concentration evaluated of 0.075 μg/mL. Flavonoids rutin and isoquercitrin were detected in 0.075 μg/mL and quantified with good precision at 0.1 μg/mL. The distinction between noise and analytical signal becomes less precise in concentrations below 0.1 μg/mL, therefore the limits of detection and quantitation determined for rutin and isoquercitrin are 0.075 and 0.1 μg/mL, respectively, shown on Table 4.

**Table 4:** Limits of detection (LOD) and quantitation (LOQ) for chlorogenic acid, rutin and isoquercitrin.

Polyphenol	LOD (μg/mL)	LOQ (μg/mL)
Chlorogenic acid	0.11	0.34
Rutin	0.075	0.1
Isoquercitrin	0.075	0.1

LOD = Limit of Detection; LOQ = Limit of Quantitation.

Other LOD and LOQ values found in the literature include 0.36 and 1.08 μg/mL [67], 14.95479 and 45.31756 μg/mL [68], respectively, for chlorogenic acid. Rutin values include LOD and LOQ of 0.09 and 0.29 μg/mL [69], and 0.19 and 0.60 μg/mL [70], respectively. Isoquercitrin LOD and LOQ values include 0.57 and 1.88 μg/mL [71], respectively. The limits of detection and quantitation found in the present study are lower than above cited, showing good sensitivity to the method in detecting and quantifying all three polyphenols.

**Fig 7:**
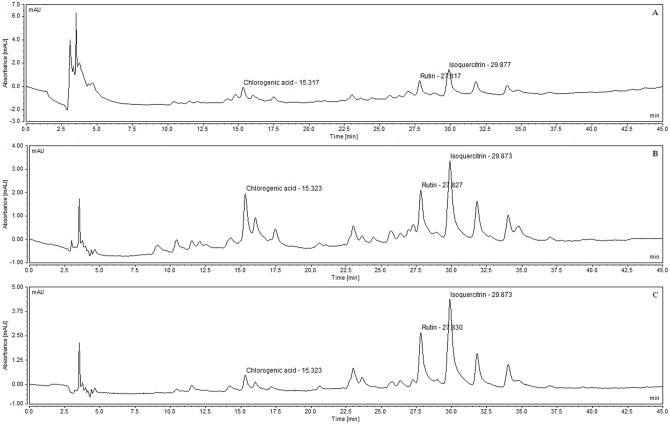
Wavelength variation on robustness. (A) 330 nm (B) 280 nm (C) 354 nm.

Wavelength changes did not lead to changes in the retention times for the three studied polyphenols. There was an already expected change in the area obtained for chlorogenic acid since it has higher absorption at 330 nm. The flavonoids rutin and isoquercitrin have higher absorption at 354 nm than at other wavelengths (280 nm and 330 nm), thereby obtaining a higher peak area in this wavelength (Fig 7). An increase in peak base width is also observed in this case, thus diminishing its resolution.

**Fig 8:**
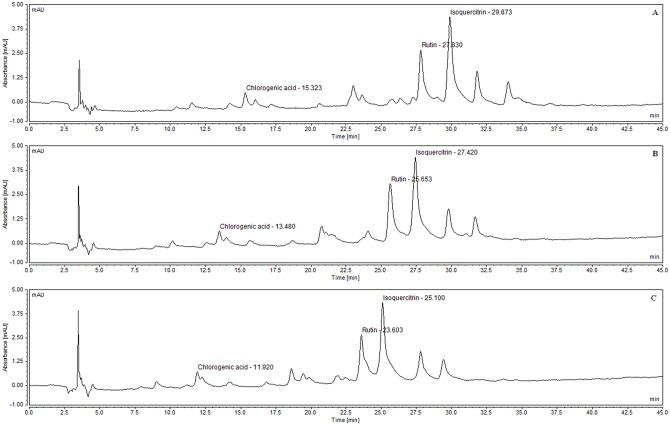
Temperature variation on robustness. (A) 25°C (B) 35°C (C) 45°C.

Increase in the column oven temperature caused displacement of the peaks, thereby altering the retention times and areas (Fig 8). Higher temperatures caused a faster elution of compounds, as expected. It was observed an increase in rutin peak area at 35°C, which can be explained by the elution of another compound in the same retention time. There was a decrease of peak area for all polyphenols when column oven temperature was 45°C.

**Fig 9:**
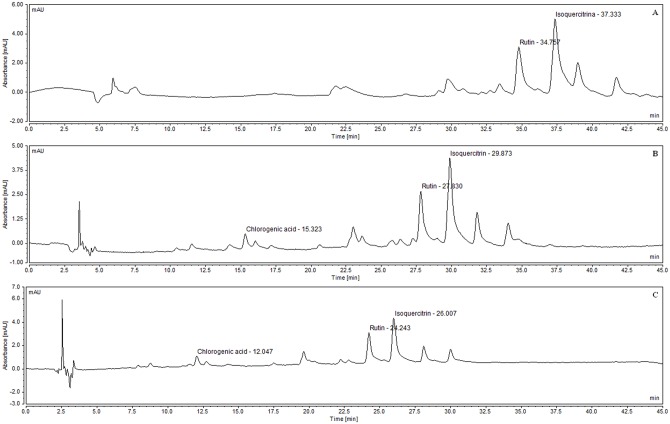
Flow variation on robustness. (A) 0.3 mL/min (B) 0.5 mL/min (C) 0.7 mL/min.

Flow change caused displacement of the peaks, thereby altering the retention times and areas (Fig 9). Higher flows caused a faster elution of compounds, as expected. Chlorogenic acid was not detected in the 0.3 mL/min flow, which makes this method less robust for this particular polyphenol, but still maintains robustness for the other parameters analyzed. It was observed an increase in peak area for all compounds when flow was slowed down to 0.3 mL/min, without altering peak purity. On the other hand, a reduction in peak area was observed for of all compounds when flow was sped up to 0.7 mL/min. The increase and decrease of peak area observed with flow reduction and elevation, respectively, are explained by the relationship between the flow rate and the area, in which the peak width will increase as the flow rate is reduced and consequently so will the peak area. Thus increasing the flow rate will result in a decrease in the response from the detector [72].

The method was robust for all three polyphenols and results are shown on Table 5.

**Table 5:** Robustness of chlorogenic acid, rutin and isoquercitrin.

		Chlorogenic acid		Rutin		Isoquercitrin	
Parameters		Retention time (min)	Area (mAU*min)	Retention time (min)	Area (mAU*min)	Retention time (min)	Area (mAU*min)
Wavelength (nm)	280	15.29 ± 0.02	0.26 ± 0.01	27.80 ± 0.02	0.42 ± 0.00	29.86 ± 0.02	0.95 ± 0.01
	330	15.30 ± 0.02	0.57 ± 0.00	27.80 ± 0.03	0.64 ± 0.01	29.85 ± 0.02	1.44 ± 0.02
	354	15.29 ± 0.03	0.17 ± 0.01	27.81 ± 0.03	0.92 ± 0.01	29.85 ± 0.02	1.98 ± 0.02
Temperature (°C)	25	15.30 ± 0.02	0.17 ± 0.01	27.81 ± 0.03	0.92 ± 0.01	29.85 ± 0.02	1.98 ± 0.02
	35	13.47 ± 0.01	0.14 ± 0.00	25.65 ± 0.01	1.03 ± 0.04	27.42 ± 0.00	1.74 ± 0.04
	45	11.90 ± 0.02	0.12 ± 0.00	23.59 ± 0.01	0.44 ± 0.01	25.09 ± 0.01	1.85 ± 0.03
Flow (mL/min)	0.3	not detected	not detected	34.75 ± 0.02	1.52 ± 0.03	37.33 ± 0.01	2.78 ± 0.03
	0.5	15.30 ± 0.02	0.17 ± 0.01	27.81 ± 0.03	0.92 ± 0.01	29.85 ± 0.02	1.98 ± 0.02
	0.7	12.05 ± 0.01	0.15 ± 0.00	24.23 ± 0.02	0.72 ± 0.01	25.98 ± 0.02	1.40 ± 0.03

The results are mean ± standard deviation of 3 experiments.

The quantitation of chlorogenic acid, rutin and isoquercitrin in all batches showed highest polyphenol content in batch 2, followed by batch 3, batch 1, batch 4 and batch 5. The polyphenols content in the extract is inversely proportional to the IC_50_ values calculated for tyrosinase inhibition. Batches with the highest concentrations of polyphenols in the extract showed the lowest IC_50_ values, as seen with batch 2 (5.14 μg/mL ± 0.24) and batch 3 (5.00 μg/mL ± 0 23). Given that the major compounds in the standardized extract of *M*. *nigra* leaves, isoquercitrin and rutin, have inhibitory activities on tyrosinase due to their metal-chelating capacities [45, 73, 74] since copper is an essential cofactor for tyrosinase activity [75], it is possible to conclude that the higher the concentration of polyphenols in the extract, greater biological activity on tyrosinase inhibition, and thus, the lower the IC_50_. According to Li et al. (2014) chlorogenic acid is probably a substrate of melanin, but their metabolic products can suppress melanogenesis in B16 melanoma cells by inhibiting tyrosinase activity [76]. Furthermore, in a study conducted by Drewa et al. (1998) mice were injected with B16 melanoma cells and treated rutin solution, wherein the administration of 10 mg of rutin inhibited melanin formation by approximately 43% [77].

The RSD for the analysis of each batch performed in triplicate was less than 15%, which indicates good accuracy as the literature does not admit values greater than 15% [66].

**Fig 10:**
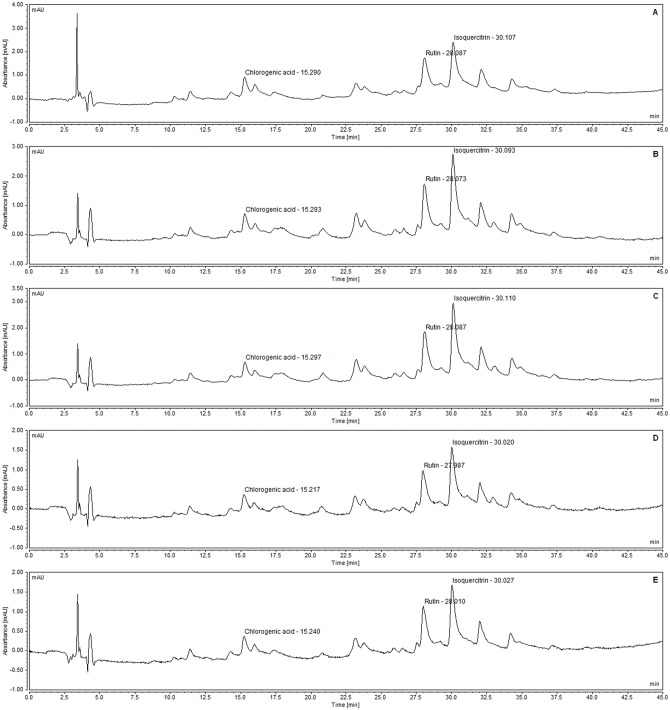
HPLC chromatogram of standardized extract of *M*. *nigra* leaves (2 mg/mL). (A) Batch 1. (B) Batch 2. (C) Batch 3. (D) Batch 4. (E) Batch 5.

It was possible to observe similar chromatographic profiles on all batches, wherein the major compound is isoquercitrin, followed by rutin. The ratios of polyphenol concentrations present in the extract were calculated. The concentration of isoquercitrin in SEML is, on average, 2.34 times greater than chlorogenic acid and, on average, 1.54 times greater than rutin in all batches (Fig 10).

**Fig 11:**
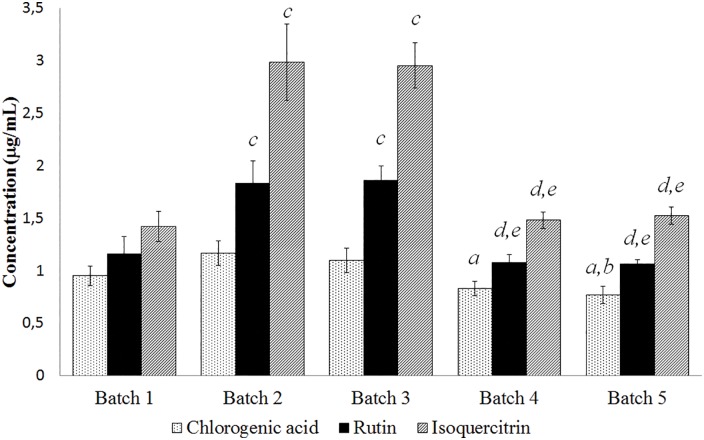
Quantification of chlorogenic acid, rutin and isoquercitrin of standardized extract of *M*. *nigra* leaves. The significant differences related to the concentration values of five batches in three experiments were calculated by one-way ANOVA with Tukey test (^*a*^*p*<0.05 *vs* Batch 2. ^b^*p*<0.05 *vs* Batch 3. ^*c*^*p*<0.05 *vs* Batch 1. ^*d*^*p*<0.05 *vs* Batch 2. ^*e*^*p*<0.05 *vs* Batch 3).

The quantification of the polyphenols in each batch was analyzed statistically by one-way ANOVA with Tukey test (*p*<0.05). There was a significant difference in the chlorogenic acid content between batch 2 with batches 4 and 5, and also significant difference between batches 3 and 5. For rutin and isoquercitrin there was significant difference between batches 2 and 3 with batches 1, 4 and 5 (Fig 11).

### Cytotoxicity analysis

**Fig 12:**
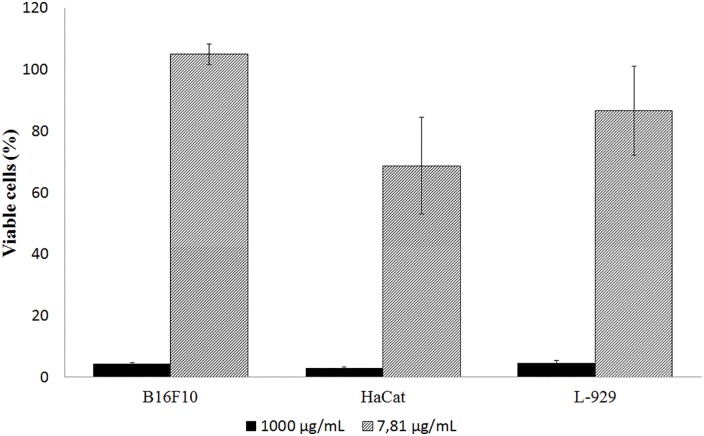
Cytotoxicity assay of melanoma (B16F10), keratinocyte (HaCat) and fibroblast (L-929) cell lines.

The cytotoxic effect of ethanol extract from leaves of *M*. *nigra* were evaluated in three cell lines, fibroblast, keratinocyte and melanoma, which take part of skin components. Treatment with SEML at the approximate tyrosinase inhibition IC_50_ value concentration (7.81 μg/mL) did not result in cell death in any cell lines, in comparison to control, after 24 hours of treatment. In all cell lines cytotoxicity was induced at the concentration of 1000 μg/mL after 24 hours of treatment, as shown in Fig 12. The solvents used in the extraction process and solubilization of the plant extract to carry out this assay, 95% ethanol and 5% methanol, respectively, showed no cytotoxic effects, thus ensuring non-interference with the cytotoxicity results. There was no interaction between SEML and WST-8 that could lead to false positive results when testing natural compounds with reducing potential inherent on the MTT tetrazolium assay. The IC_50_ values calculated for cytotoxicity were 107.2 μg/mL for murine melanoma, 324.2 μg/mL for human keratinocyte and 116.3 μg/mL for mouse fibroblast, where at these concentration values *M*. *nigra* extract showed tyrosinase inhibiting activity above 90%.

SEML was more toxic to tumor cell line of murine melanoma, suggesting that the extract has anti-tumor activity as already described in the literature by Qadir et al. (2014) in *M*. *nigra* leaf extract against human cervical cancer cell line (HeLa) [33], by Eo et al. (2014) in the study of anti-cancer activity by *M*. *alba* root bark extract on human colorectal cancer cell line (SW480) [78] and Turan et al. (2016) in the antiproliferative and apoptotic effect of *M*. *nigra* fruit extract on human prostate cancer cells (PC-3) [79].

## Conclusions

Five batches of standardized extract of *M*. *nigra* leaves were obtained, which showed reproducibility of its extraction process for total solids content, yield and moisture content. A novel source of tyrosinase inhibitor was found, which activity was also reproducible between batches. The present work developed an innovative standardization of *M*. *nigra* leaves extract using an HPLC-DAD technique for determination of polyphenols chlorogenic acid, rutin and isoquercitrin. The method validation data indicate that the method is reliable and important to the development of an herbal medicine, which serves as a tool for quality control of *M*. *nigra* extracts. The cytotoxicity results indicate that this extract is promising as a topic cosmetic due to low toxicity to cell lines that constitute the skin with high tyrosinase inhibition. The standardized extract of *M*. *nigra* leaves can be used as a potential skin depigmentation agent in the pharmaceutical and cosmetic industries and as an antibrowning agent of fruits, vegetables and beverages in the food industry.

## References

[1] Prota G. Progress in the chemistry of melanins and related metabolites. Med Res Rev. 1988;8(4):525–56. . 3057299 10.1002/med.2610080405

[2] Chang T. Natural Melanogenesis Inhibitors Acting Through the Down-Regulation of Tyrosinase Activity. Materials. 2012;5:1661–85.

[3] Wakamatsu K, Ito S. Advanced chemical methods in melanin determination. Pigment Cell Res. 2002;15(3):174–83. . 12028581 10.1034/j.1600-0749.2002.02017.x

[4] Sánchez-Ferrer A, Rodríguez-López JN, García-Cánovas F, García-Carmona F. Tyrosinase: a comprehensive review of its mechanism. Biochim Biophys Acta. 1995;1247(1):1–11. . 7873577 10.1016/0167-4838(94)00204-t

[5] Kim K. Effect of ginseng and ginsenosides on melanogenesis and their mechanism of action. J Ginseng Res. 2015;39(1):1–6. 25535470 10.1016/j.jgr.2014.10.006 PMC4268563

[6] Nole G, Johnson AW. An analysis of cumulative lifetime solar ultraviolet radiation exposure and the benefits of daily sun protection. Dermatol Ther. 2004;17 Suppl 1:57–62. . 14728700 10.1111/j.1396-0296.2004.04s1007.x

[7] Costin GE, Hearing VJ. Human skin pigmentation: melanocytes modulate skin color in response to stress. FASEB J. 2007;21(4):976–94. 10.1096/fj.06-6649rev . 17242160

[8] Lee AY, Noh M. The regulation of epidermal melanogenesis via cAMP and/or PKC signaling pathways: insights for the development of hypopigmenting agents. Arch Pharm Res. 2013;36(7):792–801. 10.1007/s12272-013-0130-6 . 23604723

[9] Pandya AG, Guevara IL. Disorders of hyperpigmentation. Dermatol Clin. 2000;18(1):91–8, ix . 10626115 10.1016/s0733-8635(05)70150-9

[10] Solano F, Briganti S, Picardo M, Ghanem G. Hypopigmenting agents: an updated review on biological, chemical and clinical aspects. Pigment Cell Res. 2006;19(6):550–71. 10.1111/j.1600-0749.2006.00334.x . 17083484

[11] Ikino JK, Nunes DH, Silva VP, Fröde TS, Sens MM. Melasma and assessment of the quality of life in Brazilian women. An Bras Dermatol. 2015;90(2):196–200. 10.1590/abd1806-4841.20152771 25830989 PMC4371668

[12] Gilchrest BA. Molecular aspects of tanning. J Invest Dermatol. 2011;131(E1):E14–7. 10.1038/skinbio.2011.6 . 22094400

[13] Kwon BS, Haq AK, Pomerantz SH, Halaban R. Isolation and sequence of a cDNA clone for human tyrosinase that maps at the mouse c-albino locus. Proc Natl Acad Sci U S A. 1987;84(21):7473–7. 2823263 10.1073/pnas.84.21.7473 PMC299318

[14] Lerner AB, Fitzpatrick TB, Calkins E, Summerson WH. Mammalian tyrosinase; preparation and properties. J Biol Chem. 1949;178(1):185–95. . 18112101

[15] Lerner AB, Fitzpatrick TB, Calkins E, Summerson WH. Mammalian tyrosinase; action on substances structurally related to tyrosine. J Biol Chem. 1951;191(2):799–806. . 14861225

[16] Mayer AM. Polyphenol oxidases in plants and fungi: going places? A review. Phytochemistry. 2006;67(21):2318–31. 10.1016/j.phytochem.2006.08.006 . 16973188

[17] Kim YJ, Uyama H. Tyrosinase inhibitors from natural and synthetic sources: structure, inhibition mechanism and perspective for the future. Cell Mol Life Sci. 2005;62(15):1707–23. 10.1007/s00018-005-5054-y . 15968468 PMC11139184

[18] Momtaz S, Lall N, Basson A. Inhibitory activities of mushroom tyrosine and DOPA oxidation by plant extracts. S Afr J Bot. 2008;74:577–82.

[19] García-Gavín J, González-Vilas D, Fernández-Redondo V, Toribio J. Pigmented contact dermatitis due to kojic acid. A paradoxical side effect of a skin lightener. Contact Dermatitis. 2010;62(1):63–4. 10.1111/j.1600-0536.2009.01673.x . 20136888

[20] Curto EV, Kwong C, Hermersdörfer H, Glatt H, Santis C, Virador V, et al Inhibitors of mammalian melanocyte tyrosinase: *in vitro* comparisons of alkyl esters of gentisic acid with other putative inhibitors. Biochem Pharmacol. 1999;57(6):663–72. . 10037452 10.1016/s0006-2952(98)00340-2

[21] Zhu W, Gao J. The use of botanical extracts as topical skin-lightening agents for the improvement of skin pigmentation disorders. J Investig Dermatol Symp Proc. 2008;13(1):20–4. 10.1038/jidsymp.2008.8 . 18369335

[22] Ribas J, Schettini APM, Cavalcante MdSM. Ocronose exógena induzida por hidroquinona: relato de quatro casos. An Bras Dermatol. 2010;85.

[23] Gandhi V, Verma P, Naik G. Exogenous ochronosis After Prolonged Use of Topical Hydroquinone (2%) in a 50-Year-Old Indian Female. Indian J Dermatol. 2012;57(5):394–5. 10.4103/0019-5154.100498 23112363 PMC3482806

[24] Zheng Z-P, Cheng K-W, Chao J, Wu J, Wang M. Tyrosinase inhibitors from paper mulberry (*Broussonetia papyrifera*). Food Chem. 2008;106:529–35.

[25] Souza PM, Elias ST, Simeoni LA, de Paula JE, Gomes SM, Guerra EN, et al Plants from Brazilian Cerrado with potent tyrosinase inhibitory activity. PLoS One. 2012;7(11):e48589 10.1371/journal.pone.0048589 23173036 PMC3500240

[26] Son SM, Moon KD, Lee CY. Rhubarb Juice as a Natural Antibrowning Agent. J Food Sci. 2000;65(8):1288–9.

[27] Watson L, Dallwitz MJ. The families of flowering plants: descriptions, illustrations, identification, and information retrieval 1992 [cited 15 July 2015]. Available: delta-intkey.com.

[28] Özgen M, Serçe S, Kaya C. Phytochemical and antioxidant properties of anthocyanin-rich *Morus nigra* and *Morus rubra* fruits. Sci Hortic (Amsterdam). 2009;119(3):275–9.

[29] Wang S, Liu XM, Zhang J, Zhang YQ. An efficient preparation of mulberroside a from the branch bark of mulberry and its effect on the inhibition of tyrosinase activity. PLoS One. 2014;9(10):e109396 10.1371/journal.pone.0109396 25299075 PMC4192315

[30] Hanif F, Singh DK. Molluscicidal activity of *Morus nigra* against the freshwater snail *Lymnaea acuminata*. J biol earth sci. 2012;2(2):B54–B62.

[31] Oliveira ACB, Oliveira AP, Guimarães AL, Oliveira RA, Silva FS, Reirs SAGB, et al Avaliação toxicológica pré-clínica do chá das folhas de *Morus nigra* L. (Moraceae). Rev Bras Pl Med. 2013;15(2):244–9.

[32] Mazimba O, Majinda RRT, Motlhanka D. Antioxidant and antibacterial constituents from *Morus nigra*. Afr J Pharm Pharmaco. 2011;5(6):751–4.

[33] Qadir MI, Ali M, Ibrahim Z. Anticancer activity of *Morus nigra* leaves extract. Bangladesh J Pharmacol. 2014;9:496–7.

[34] Mallhi TH, Qadir MI, Khan YH, Ali M. Hepatoprotective activity of aqueous methanolic extract of *Morus nigra* against paracetamol-induced hepatotoxicity in mice. Bangladesh J Pharmacol. 2014;9:60–6.

[35] Tag HM. Hepatoprotective effect of mulberry (*Morus nigra*) leaves extract against methotrexate induced hepatotoxicity in male albino rat. BMC Complement Altern Med. 2015;15:252 10.1186/s12906-015-0744-y 26209437 PMC4514987

[36] Naderi GA, Asgary S, Sarraf-Zadegan N, Oroojy H, Afshin-Nia F. Antioxidant activity of three extracts of *Morus nigra*. Phytother Res. 2004;18(5):365–9. 10.1002/ptr.1400 . 15173994

[37] Chen H, Pu J, Liu D, Yu W, Shao Y, Yang G, et al Anti-Inflammatory and Antinociceptive Properties of Flavonoids from the Fruits of Black Mulberry (*Morus nigra* L.). PLoS One. 2016;11(4):e0153080 10.1371/journal.pone.0153080 27046026 PMC4821529

[38] Ercisli S, Orhan E. Chemical composition of white (*Morus alba*), red (*Morus rubra*) and black (*Morus nigra*) mulberry fruits. Food Chem. 2007;103:1380–4.

[39] Ercisli S, Tosun M, Duralija B, Voca S, Sengul M, Turan M. Phytochemical Content of Some Black (*Morus nigra* L.) and Purple (*Morus rubra* L.) Mulberry Genotypes. Food Technol Biotechnol. 2010;48(1):102–6.

[40] Arfan M, Khan R, Rybarczyk A, Amarowicz R. Antioxidant activity of mulberry fruit extracts. Int J Mol Sci. 2012;13(2):2472–80. 10.3390/ijms13022472 22408465 PMC3292034

[41] Jiang Y, Nie WJ. Chemical properties in fruits of mulberry species from the Xinjiang province of China. Food Chem. 2015;174:460–6. 10.1016/j.foodchem.2014.11.083 . 25529706

[42] Chang TS. An updated review of tyrosinase inhibitors. Int J Mol Sci. 2009;10(6):2440–75. 10.3390/ijms10062440 19582213 PMC2705500

[43] Park KT, Kim JK, Hwang D, Yoo Y, Lim YH. Inhibitory effect of mulberroside A and its derivatives on melanogenesis induced by ultraviolet B irradiation. Food Chem Toxicol. 2011;49(12):3038–45. 10.1016/j.fct.2011.09.008 . 21946069

[44] Lee SH, Choi SY, Kim H, Hwang JS, Lee BG, Gao JJ, et al Mulberroside F isolated from the leaves of *Morus alba* inhibits melanin biosynthesis. Biol Pharm Bull. 2002;25(8):1045–8. . 12186407 10.1248/bpb.25.1045

[45] Chang LW, Juang LJ, Wang BS, Wang MY, Tai HM, Hung WJ, et al Antioxidant and antityrosinase activity of mulberry (*Morus alba* L.) twigs and root bark. Food Chem Toxicol. 2011;49(4):785–90. 10.1016/j.fct.2010.11.045 . 21130832

[46] Zheng ZP, Tan HY, Wang M. Tyrosinase inhibition constituents from the roots of *Morus australis*. Fitoterapia. 2012;83(6):1008–13. 10.1016/j.fitote.2012.06.001 . 22698714

[47] Takahashi M, Takara K, Toyozato T, Wada K. A novel bioactive chalcone of *Morus australis* inhibits tyrosinase activity and melanin biosynthesis in B16 melanoma cells. J Oleo Sci. 2012;61(10):585–92. . 23018855 10.5650/jos.61.585

[48] Masuda T, Yamashita D, Takeda Y, Yonemori S. Screening for Tyrosinase Inhibitors among Extracts of Seashore Plants and Identification of Potent Inhibitors from *Gardenia subelliptica*. Biosci Biotechnol Biochem. 2005;69(1):197–201. 15665485 10.1271/bbb.69.197

[49] Ryu YB, Ha TJ, Curtis-Long MJ, Ryu HW, Gal SW, Park KH. Inhibitory effects on mushroom tyrosinase by flavones from the stem barks of *Morus lhou* (S.) Koidz. J Enzyme Inhib Med Chem. 2008;23(6):922–30. 10.1080/14756360701810207 . 18608767

[50] Jeong SH, Ryu YB, Curtis-Long MJ, Ryu HW, Baek YS, Kang JE, et al Tyrosinase inhibitory polyphenols from roots of *Morus lhou*. J Agric Food Chem. 2009;57(4):1195–203. 10.1021/jf8033286 . 19166303

[51] Hu X, Wang M, Yan GR, Yu MH, Wang HY, Hou AJ. 2-Arylbenzofuran and tyrosinase inhibitory constituents of *Morus notabilis*. J Asian Nat Prod Res. 2012;14(12):1103–8. 10.1080/10286020.2012.724400 . 23088613

[52] Hu X, Wu JW, Wang M, Yu MH, Zhao QS, Wang HY, et al 2-Arylbenzofuran, flavonoid, and tyrosinase inhibitory constituents of *Morus yunnanensis*. J Nat Prod. 2012;75(1):82–7. 10.1021/np2007318 . 22165973

[53] Zhang X, Hu X, Hou A, Wang H. Inhibitory effect of 2,4,2',4'-tetrahydroxy-3-(3-methyl-2-butenyl)-chalcone on tyrosinase activity and melanin biosynthesis. Biol Pharm Bull. 2009;32(1):86–90. . 19122286 10.1248/bpb.32.86

[54] Zheng ZP, Cheng KW, Zhu Q, Wang XC, Lin ZX, Wang M. Tyrosinase inhibitory constituents from the roots of *Morus nigra*: a structure-activity relationship study. J Agric Food Chem. 2010;58(9):5368–73. 10.1021/jf1003607 . 20297841

[55] Eleazu CO, Eleazu KC, A E., Chukwuma SC. Comparative study of the phytochemical composition of the leaves of five Nigerian medicinal plants. Journal of Biotechnology and Pharmaceutical Research. 2012;3(2):42–6.

[56] Patel DK, Patel K, Duraiswamy B, Dhanabal SP. Phytochemical analysis and standardization of *Strychnos nux-vomica* extract through HPTLC techniques. Asian Pac J Trop Dis. 2012:S56–S60.

[57] Ravi A, Mallika A, Sama V, Begum AS, Khan RS, Reddy BM. Antiproliferative activity and standardization of *Tecomella undulata* bark extract on K562 cells. J Ethnopharmacol. 2011;137:1353–9. 10.1016/j.jep.2011.07.067 . 21843623

[58] Palav Y, D'mello PM. Standardization of selected Indian medicinal herbal raw materials containing polyphenols as major phytoconstituents. Indian J Pharm Sci. 2006;68(4):506–9.

[59] Garg V, Dhar VJ, Sharma A, Dutt R. Facts about standardization of herbal medicine: a review. Zhong Xi Yi Jie He Xue Bao. 2012;10(10):1077–83. . 23073189 10.3736/jcim20121002

[60] Khatib S, Nerya O, Musa R, Shmuel M, Tamir S, Vaya J. Chalcones as potent tyrosinase inhibitors: the importance of a 2,4-substituted resorcinol moiety. Bioorg Med Chem. 2005;13(2):433–41. 10.1016/j.bmc.2004.10.010 . 15598564

[61] ICH. Guidance for Industry. Q2B Validation of Analytical Procedures: Methodology. 1996.

[62] Agência Nacional de Vigilância Sanitária (Brasil). Resolução—RE N° 899, de 29 de maio de 2003. Guia para Validação de Métodos Analíticos e Bioanalíticos. 2003.

[63] Sánchez-Salcedo EM, Tassotti M, Del Rio D, Hernández F, Martínez JJ, Mena P. (Poly)phenolic fingerprint and chemometric analysis of white (*Morus alba* L.) and black (*Morus nigra* L.) mulberry leaves by using a non-targeted UHPLC-MS approach. Food Chem. 2016;212:250–5. 10.1016/j.foodchem.2016.05.121 . 27374530

[64] Sánchez-Salcedo EM, Mena P, García-Veguera C, Hernández F, Martínez JJ. (Poly)phenolic compounds and antioxidant activity of white (*Morus alba*) and black (*Morus nigra*) mulberry leaves: Their potential for new products rich in phytochemicals. J Funct Foods. 2015;18(Part B):1039–46.

[65] Hunyadi A, Martins A, Hsieh TJ, Seres A, Zupkó I. Chlorogenic acid and rutin play a major role in the in vivo anti-diabetic activity of *Morus alba* leaf extract on type II diabetic rats. PLoS One. 2012;7(11):e50619 10.1371/journal.pone.0050619 23185641 PMC3503931

[66] Agência Nacional de Vigilância Sanitária (Brasil). Instrução Normativa N° 4, de 18 de junho de 2014. Guia de orientação para registro de Medicamento Fitoteraápico e registro e notificação de Produto Tradicional Fitoteraápico. Diário Oficial da União 2 jun 2014.

[67] Şarer E, Gokbulut A. Determination of caffeic and chlorogenic acids in the leaves and fruits of *Vitex agnus-castus*. Turkish Journal of Pharmaceutical Sciences. 2008;5(3):167–74.

[68] Adham AN. Simultaneous estimation of caffeic and chlorogenic acid content in *Ammi majus* seed by TLCA and HPLC. Int J Pharm Pharm Sci. 2015;7(6):263–7.

[69] Landim LP, Feitoza GS, Costa JGM. Development and validation of a HPLC method for the quantification of three flavonoids in a crude extract of *Dimorphandra gardneriana*. Rev Bras Farmacogn. 2013;23(1):58–64.

[70] Leite CFM, Leite BHM, Barros IMdC, Gomes SM, Fagg CW, Simeoni LA, et al Determination of rutin in *Erythroxylum suberosum* extract by liquid chromatography: applicability in standardization of herbs and stability studies. Bol Latinoam Caribe. 2014;13(2):135–43.

[71] Dutra DM, Barth CdS, Block LC, Quintão NLM, Couto AG, Cechinel Filho V, et al Simultaneous determination of four phenolic compounds in extracts of aereal parts of *Ipomoea pes-caprae* (L.) R. Br. (Convolvulaceae) by HPLC-UV. Quim Nova. 2014;37(9):1510–4.

[72] Today Chromatography. Why Is My Peak Area Reducing With Flow Rate? 2014 Nov 26 [cited 17 December 2015]. Available: http://www.chromatographytoday.com/articles/bioanalytical/40/chromatography_today_help_desk/why_is_my_peak_area_reducing_with_flow_rate/1750/%5D.

[73] Si YX, Yin SJ, Oh S, Wang ZJ, Ye S, Yan L, et al An integrated study of tyrosinase inhibition by rutin: progress using a computational simulation. J Biomol Struct Dyn. 2012;29(5):999–1012. 10.1080/073911012010525028 . 22292957

[74] Ziaullah, Bhullar KS, Warnakulasuriya SN, Rupasinghe HP. Biocatalytic synthesis, structural elucidation, antioxidant capacity and tyrosinase inhibition activity of long chain fatty acid acylated derivatives of phloridzin and isoquercitrin. Bioorg Med Chem. 2013;21(3):684–92. 10.1016/j.bmc.2012.11.034 . 23266182

[75] Lerch K, Huber M, Schneider H-J, Drexel R, Linzen B. Different Origins of Metal Binding Sites in Binuclear Copper Proteins, Tyrosinase and Hemocyanin. J Inorg Biochem. 1986;26(3):213–7.

[76] Li HR, Habasi M, Xie LZ, Aisa HA. Effect of chlorogenic acid on melanogenesis of B16 melanoma cells. Molecules. 2014;19(9):12940–8. 10.3390/molecules190912940 . 25157464 PMC6271456

[77] Drewa G, Schachtschabel DO, Pałgan K, Grzanka A, Sujkowska R. The influence of rutin on the weight, metastasis and melanin content of B16 melanotic melanoma in C57BL/6 mice. Neoplasma. 1998;45(4):266–71. . 9890672

[78] Eo HJ, Park JH, Park GH, Lee MH, Lee JR, Koo JS, et al Anti-inflammatory and anti-cancer activity of mulberry (*Morus alba* L.) root bark. BMC Complement Altern Med. 2014;14:200 10.1186/1472-6882-14-200 24962785 PMC4074313

[79] Turan I, Demir S, Kilinc K, Burnaz NA, Yaman SO, Akbulut K, et al Antiproliferative and apoptotic effect of *Morus nigra* extract on human prostate cancer cells. Saudi Pharm J. 2016. In Press. 10.1016/j.jsps.2016.06.002 PMC5355563 28344475

